# The mechanistic immunosuppressive role of the tumour vasculature and potential nanoparticle-mediated therapeutic strategies

**DOI:** 10.3389/fimmu.2022.976677

**Published:** 2022-08-15

**Authors:** Zakaria Elias Ileiwat, Tanveer A. Tabish, Dmitry A. Zinovkin, Jale Yuzugulen, Nahid Arghiani, Md Zahidul I. Pranjol

**Affiliations:** ^1^ Department of Biochemistry and Biomedicine, School of Life Sciences, University of Sussex, Brighton, United Kingdom; ^2^ Division of Cardiovascular Medicine, Radcliffe Department of Medicine, University of Oxford, Oxford, United Kingdom; ^3^ Department of Pathology, Gomel State Medical University, Gomel, Belarus; ^4^ Faculty of Pharmacy, Eastern Mediterranean University, Famagusta, Cyprus; ^5^ Department of Molecular Biosciences, The Wenner-Gren Institute, Stockholm University, Stockholm, Sweden

**Keywords:** angiogenesis, endothelium, immunosuppression, vascular normalisation, nanotherapy

## Abstract

The tumour vasculature is well-established to display irregular structure and hierarchy that is conducive to promoting tumour growth and metastasis while maintaining immunosuppression. As tumours grow, their metabolic rate increases while their distance from blood vessels furthers, generating a hypoxic and acidic tumour microenvironment. Consequently, cancer cells upregulate the expression of pro-angiogenic factors which propagate aberrant blood vessel formation. This generates atypical vascular features that reduce chemotherapy, radiotherapy, and immunotherapy efficacy. Therefore, the development of therapies aiming to restore the vasculature to a functional state remains a necessary research target. Many anti-angiogenic therapies aim to target this such as bevacizumab or sunitinib but have shown variable efficacy in solid tumours due to intrinsic or acquired resistance. Therefore, novel therapeutic strategies such as combination therapies and nanotechnology-mediated therapies may provide alternatives to overcoming the barriers generated by the tumour vasculature. This review summarises the mechanisms that induce abnormal tumour angiogenesis and how the vasculature’s features elicit immunosuppression. Furthermore, the review explores examples of treatment regiments that target the tumour vasculature.

## Introduction

Angiogenesis is the process of new blood vessel formation from existing vasculature and is abundant in growing tumours (1). As tumours increase in size, the accompanying increased demand for nutrients and oxygen upregulates the excess production of pro-angiogenic factors such as vascular endothelial growth factor (VEGF) and basic fibroblast growth factor (bFGF) from tumour cells, termed the ‘angiogenic switch’ (2). However, the new vasculature often displays increased permeability, tortuosity, and immaturity, thus facilitating metastasis but not supplying the metabolic demands of the tumour (3, 4). These abnormal vessels are also poorly perfused due to a lack of mural cell recruitment and basement membrane coverage, leading to increased interstitial pressure (5, 6). These features ultimately form areas of hypoxia that generate an immunosuppressive environment by inhibiting effector T cell infiltration while upregulating the presence of immunosuppressive cells such as myeloid-derived suppressor cells (MDSCs) and regulatory T cells (Tregs) (1, 6, 7).

Importantly, the tumour vasculature can attenuate chemotherapeutic drug delivery or prevent the crucial formation of reactive oxygen species during radiotherapy (8). Therefore, treatments aiming to return the vasculature to a physiologically functional state termed ‘vascular normalisation’ are ideal areas of cancer research (6). Nonetheless, current anti-angiogenic drugs such as bevacizumab and sunitinib have shown variable successes in solid tumours, due to focusing largely on the effects of VEGF (9). However, the emerging use of nanoparticle technology in cancer therapy has shown promise in improving the efficacy of anti-angiogenic and chemotherapeutic drug treatments by enhancing their pharmacokinetic properties (9). Moreover, the ability to modify these nanoparticles allows for targeted delivery which can be employed to alleviate anti-cancer immunosuppression such as *via* endothelial cell regulation (10).

This review will therefore summarise the mechanisms leading to the immunosuppressive tumour microenvironment (TME) resulting from the generation of the tumour vasculature’s features. In addition, this review will discuss examples of past and novel treatments that target the aberrant tumour vasculature, promote normalisation, and improve traditional cancer therapies.

## Angiogenesis and the structure of the tumour vasculature

### Mechanisms maintaining blood supply in tumours

**Figure 1:**
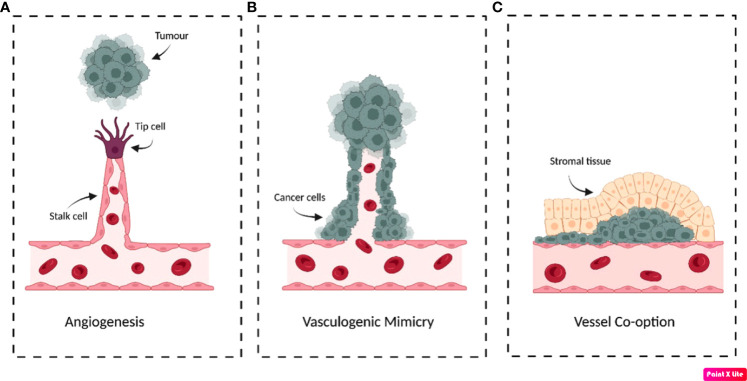
Schematic illustration demonstrating the possible mechanisms that tumours use to obtain blood supply. **(A)** Sprouting angiogenesis. In response to pro-angiogenic factors secreted by tumours, endothelial tip cells form that migrate towards the tumour’s growth factor signalling. **(B)** Vasculogenic/vascular mimicry. Often in response to hypoxia, cancer cells differentiate into endothelial-like phenotypes to form tubular structures that resemble blood vessels to support the tumour. **(C)** Vessel co-option. Cancer cells migrate along the pre-existing vessel that supplies the tissue parenchyma. Cancer cells can form circular cuffs that surround the vessel and/or invade the surrounding stroma to expropriate the tissue blood supply. Original figure created with BioRender.com.

Tumours primarily induce new vessel formation *via* sprouting angiogenesis, forming most of the abnormal vasculature (**Figure 1A**) (11). Physiologically, angiogenesis is well-coordinated but due to the angiogenic switch, the process is highly dysregulated. Secretion of pro-angiogenic factors such as VEGF-A by tumours initiates the process, weakening endothelial cell junctions and causing vessel-pericyte dissociation (11). Thereafter, proteases such as matrix metalloproteinases (MMPs) and cathepsins degrade the endothelial basement membrane (11, 12). VEGF-A then triggers angiogenic sprouting, creating tip cells that direct new vessel formation and induce stalk cell proliferation to form the vascular lumen (13–15). Lastly, pericytes are recruited by factors such as angiopoietin-1 (Ang-1) and platelet-derived growth factor-β (PDGF-β) to stabilise the new vessel (16).

Despite this, tumours have developed the ability to induce non-angiogenic neovascularisations by forming tubular structures composed of cancer cells termed ‘vasculogenic/vascular mimicry’ (VM) (**Figure 1B**) (17). Tumours displaying VM have been increasingly linked to enhanced aggressiveness and invasiveness, demonstrated in a variety of cancers such as prostate, breast and gastrointestinal tumours (17). For example, in patients suffering from gastrointestinal tumours, VM was significantly more prevalent in tumours classified as high-risk than very-low/low-risk, where incidence was 39.5% and 5.9%, respectively (17). Currently, the criteria distinguishing VM from other vascularisation mechanisms necessitates that the vessels be composed of cells that are positive for periodic acid-Schiff (PAS) staining and negative for immunohistochemical detection of endothelial marker CD31 (18). Additionally, these structures must have erythrocytes present in the vessel lumen to be characterised as VM vascularisations (18). The mechanisms initiating VM remain to be fully elucidated, but evidence has suggested hypoxia/hypoxia-inducible factors (HIF) to be major inducers that could even result from anti-angiogenic therapy (19, 20).

‘Vessel co-option’ is an alternative mechanism for maintaining blood supply by utilising existing vasculature without inducing *de novo* vessel formation (21). This is often histologically identified by the presence of cancer cells surrounding structurally and architecturally functional vessels, contrary to the abnormal vasculature observed in angiogenesis (**Figure 1C**) (21). This has been observed in metastatic and primary tumours of the lung, brain and liver and may contribute to intrinsic and acquired anti-VEGF therapy resistance (22–24). This was observed in hepatocellular carcinoma xenografts, which displayed a statistically significant 51.7% increase in the proportion of co-opted vessels between untreated control and sorafenib resistant tumours (24). Similarly, lung metastasis models treated with sunitinib demonstrated altered growth patterns that promoted vessel co-option, further demonstrating an alternative mechanism of anti-angiogenic therapy resistance (25).

### Features of the tumour vasculature

**Figure 2:**
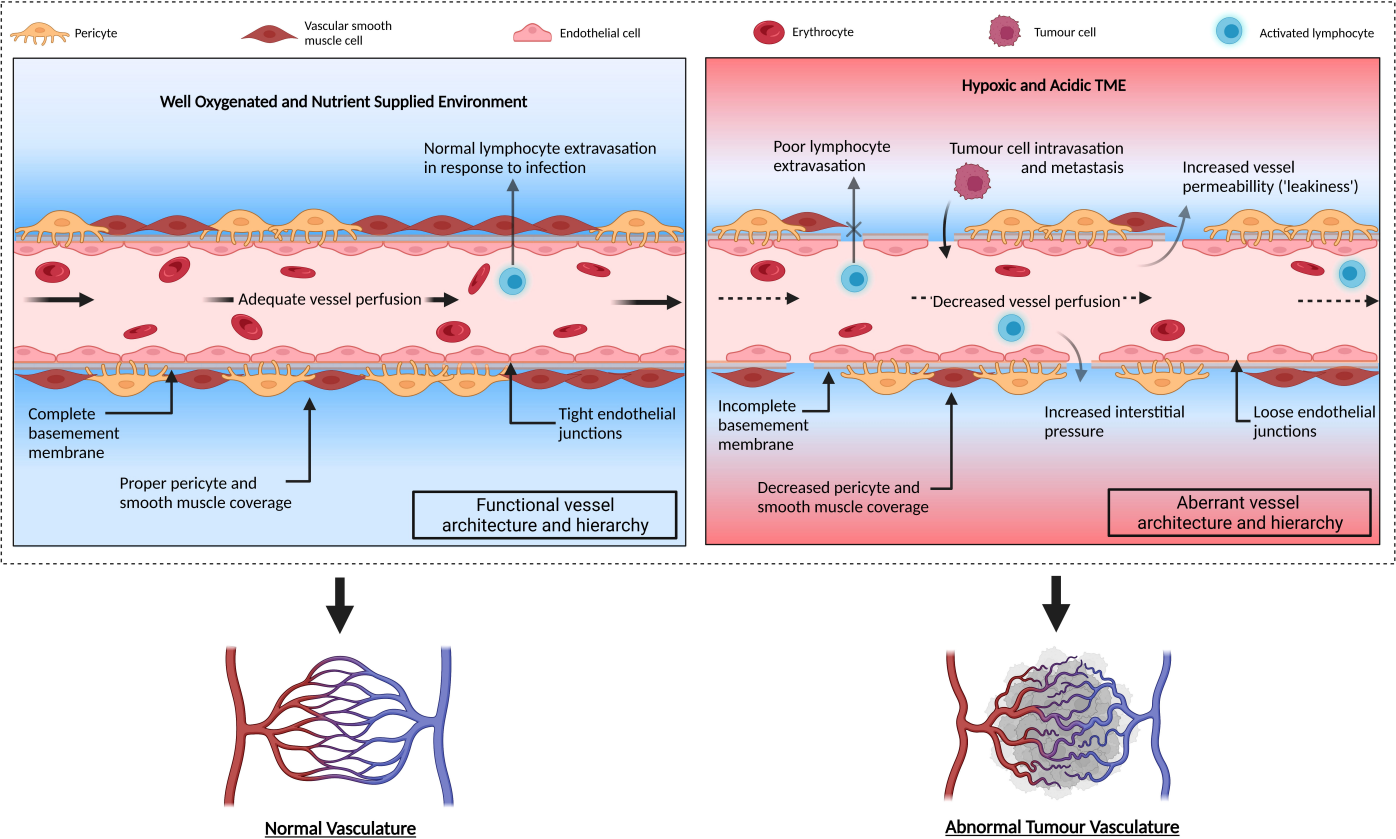
Schematic illustration of normal functioning vasculature compared to aberrant tumour vasculature. Normal vessels demonstrate correct vascular hierarchy composed of arteries, arterioles, capillaries, venules, and veins. The vasculature is well-organised with endothelial cells exhibiting complete basement membrane and adequate mural cell (pericyte and vascular smooth muscle cell) coverage. The vessels are well perfused allowing sufficient oxygen supply and metabolic waste removal from the surrounding tissue. Conversely, the tumour vasculature exhibits reduced effector T cell extravasation into tumour parenchyma and displays a lack of mural cell coverage which destabilises the vessel. The relaxed endothelial tight junctions and incomplete basement membrane coverage allows fluid to escape into the surrounding area, increasing the surrounding interstitial pressure and reducing vessel perfusion. This prevents adequate oxygen supply to the tissues and ineffectively removes metabolic waste. Consequently, the surrounding area becomes hypoxic and acidic. Original figure created with BioRender.com.

Tumour vessels are structurally and functionally heterogeneous with various cellular and molecular abnormalities (**Figure 2**). Mural cell coverage has been linked to promoting vessel stabilisation and endothelial cell survival while inhibiting endothelial cell proliferation (26). Recruitment of these cells is mostly mediated by PDGF-β secreted by endothelial cells but *in situ* hybridisation from mouse tumour models demonstrated that endothelial cells did not lack PDGF-β expression (26). This suggests that mural cell recruitment defects may be due to a lack of other contributing factors such as a decreased pool of mural cell progenitors (26). In addition, vascular hyperpermeability has been associated with upregulated angiogenesis and may generate aberrant vasculature with an increased dependence on VEGF-A for survival (5, 26). These ‘leaky’ vessels can lead to increased interstitial pressure, blood viscosity and large protein extravasation from the vasculature (4, 5). In addition, tumour blood vessels often display poor vascular perfusion that can inhibit normal function and delivery of chemotherapeutic drugs (27). Evidence has shown this could result from increased tumour growth that causes physical compression and subsequent collapse of intratumoral vessels (27).

### The angiogenic switch

As solid tumours develop in size past 1-2 millimetres, the radial diffusion distance from the associated blood vessels increases (1, 28). As the tumour is unable to receive adequate oxygen and nutrient supply, hypoxic areas form which promote angiogenesis (28). These hypoxic conditions stimulate HIF-1 and HIF-2 formation, which induces the expression of proangiogenic factors required for new vessel formation such as VEGF, PDGF-β, Ang-1 and Ang-2 (29, 30). However, the established imbalance of excess pro-angiogenic factors to anti-angiogenic factors continues generating aberrant vasculature that does not deliver sufficient oxygen supply. This perpetuates the presence of hypoxic and acidic areas, prompting these tumours to have poor prognoses (31).

VEGF, Ang-1 and Ang-2 are the main growth factors responsible for tumour angiogenesis indicated by their essentially restricted receptor expression on endothelial cells (32). When acting solitarily, Ang-2 can antagonise the stabilising function of Ang-1 by blocking the Tie2 receptor (33). This promotes vessel regression and remodelling; however, co-expression of VEGF and Ang-2 has been shown to have a pro-angiogenic effect on endothelial cells, suggesting a role in tumour vasculature formation (33). For example, hepatocellular carcinoma specimens expressing both Ang-2 and VEGF demonstrated increased microvessel density, vessel destabilisation and tumour size (32, 33). Furthermore, hepatocellular carcinoma specimens positive for Ang-2 mRNA expression displayed an increased tumour microvessel area compared to their Ang-2 mRNA negative counterparts (32). In addition, Ang-2 overexpression was linked to endothelial cell aggregation, vessel leakage and decreased vessel lumen size which may lead to hypoxic regions, further promoting angiogenesis (34).

### The tumour microenvironment and hypoxia induced extracellular matrix remodelling

The ECM is a major component of the TME and is primarily composed of collagen and fibronectin (35). However, alterations to the ECM composition can cause the release of sequestered pro-angiogenic factors that facilitate angiogenesis (36). Under hypoxic conditions, endothelial cells increase the deposition of ECM components such as fibronectin, collagen IV and laminin which have been demonstrated to promote endothelial cell proliferation, elongation, migration, and survival necessary for angiogenesis (36).

In response to HIF-1, fibroblasts display upregulation of collagen prolyl 4-hydroxylases such as P4HA1 and P4HA2 as well as collagen lysyl hydroxylases such as procollagen-lysine, 2-oxoglutarate 5-dioxygenase 2 (PLOD2) (35, 37). During 24-hour exposure to hypoxic 1% O_2_ conditions, mRNA expression of P4HA1, P4HA2, and PLOD2 in fibroblasts had increased over 5 times compared to fibroblasts exposed to 20% O_2_ conditions (35). These enzymes aid in collagen fibre formation that stiffens the ECM. P4HA1 and P4HA2 accomplish this by increasing collagen cross-linking while PLOD2 increases collagen deposition (35). This promotes cancer cell invasiveness and disrupts tumour endothelial cell alignment (35, 38). Experiments revealed these tumour endothelial cells exhibited increased constitutive Rho and ROCK activity, therefore increasing cytoskeletal tension which impedes endothelial cell mechanosensitivity (38). Tumour capillary endothelial cells displayed 2.5 times greater baseline Rho activity and 4 times greater baseline ROCK activity compared to normal endothelial cells (38). Increased ECM stiffness was suggested to trigger this, which consequently increased the formation of capillary networks and aberrant vascular structures (38).

In addition, in response to hypoxia, ECM composition can, directly and indirectly, affect protease expression. When grown on a collagen I gel matrix compared to a Matrigel matrix, rat endothelial cells displayed upregulated MT1-MMP and MMP2 expression, possibly resulting from ligand binding or the aforementioned aberrant endothelial mechanosensory mechanism (36, 39). MMPs are vital during angiogenesis as they degrade the main components of endothelial basement membranes (40). These MMPs expressed by tumour cells, fibroblasts and endothelial cells can digest various ECM components, further promoting tumour invasion and metastasis (40, 41). Crucially, MMP2 and MMP9 expression is often upregulated in tumours and their degradation of components like proteoglycans can release bound bioactive components termed matrikines or matricryptins (42, 43). These matrikines induce MMP expression *via* positive feedback loops, such as canstatin release increases MMP2 and MMP9 expression in fibroblasts (42, 43). These MMPs promote vascular and tumoral invasion as well as release tumour necrosis factor-alpha (TNF-α) and soluble Fas ligand which inhibits tumour cell apoptosis (41). Data showed that MMP2 and MMP9 expression was positively correlated to tumour invasion depth, venous invasion and increased tumour size (over 4cm) (41). Crucially, MT1-MMP and MMP9 activity can release extracellular heparin-bound VEGF which can induce MMP2 and MMP9 expression from tumour cells, likely *via* VEGFR2 activation (44–46).

Cathepsins including cathepsin L, B and D are other ECM proteases exhibiting pro-angiogenic action and upregulation in many tumours (47–55). Upregulation of cathepsin L *via* fibroblast growth factor (FGF) and VEGF causes the enzyme to be extracellularly secreted as opposed to its normal function within lysosomes (47). *In vitro* studies showed cathepsin L’s paracrine interaction with endothelial cells increases endothelial invasion, migration and sprouting while cathepsin L inhibition suppresses angiogenesis in xenograft breast cancer models (47). Moreover, cathepsin L-induced galectin-1 has been shown to induce proliferation and migration microvascular endothelial cells, demonstrating a proangiogenic role of this lysosomal enzyme (55). Additionally, cathepsin B can degrade ECM laminin, collagen IV and fibronectin as well as induce pro-urokinase-type plasminogen activator (pro-uPA) activation in xenograft glioblastoma models (48, 56). This upregulates VEGF expression in tumours, thus inducing angiogenesis (48). Lastly, cathepsin D upregulation *via* HIF-1α is suggested to promote angiogenesis by activating cathepsin B and proteolytically releasing bFGF (57, 58). However, both Cathepsin D and L have been shown to enhance proliferation and migration of microvascular endothelial cells in a non-proteolytic manner, leaving its angiogenic mechanisms unresolved (50, 51, 57).

Tumour-associated macrophages (TAMs) are another component of the TME and have been shown to induce ECM remodelling by expressing a range of enzymes such as collagen prolyl 4-hydroxylases, collagen lysyl hydroxylases, MMPs (including MT1-MMP), a disintegrin and metalloproteinases (ADAMs) and cathepsins that aid angiogenesis (59). Besides expressing ECM remodelling enzymes, TAMs can also modify the enzymatic activity of other ECM remodelling cells such as by expressing procollagen c-endopeptidase enhancer (PCOLCE) to upregulate procollagen C–proteinase’s collagen maturation activity (59). This demonstrates TAMs unique ability to affect ECM remodelling in a manner typically associated with fibroblasts (59).

One of the most important components of the TME are fibroblasts which have a primary role in synthesising collagen and are essential in maintaining the EMC structure of associated tissues (60). However, cancer-associated fibroblasts (CAFs) which are located within or near tumours may have altered metabolism and function causing them to secrete factors, chemokines, and enzymes such as VEGFA, CXCL12 and MMPs that can promote angiogenesis (60). Preclinical evidence has also suggested that certain CAFs may have a role in reducing immunotherapy efficacy, making it another possible target of cancer therapy (60). Similar to CAFs, adipocytes can also secrete pro-angiogenic factors, chemokines and cytokines known as adipokines which include TNF-α, VEGF-A and FGF2 (37, 61). Additionally, adipocytes can support angiogenesis by releasing fatty acids during lipolysis which is upregulated by tumour-released factors. The resulting increased fatty acid availability promotes β-oxidation in endothelial cells, encouraging angiogenesis (37).

## The Immunosuppressive role of the tumour vasculature

Tumour vasculature plays key roles in immunosuppression through various mechanisms, many of which have been reviewed elsewhere (62–64). In the following sections we describe mechanistic roles of endothelial cells and tumour vascular-associated ECM components in suppressing an anti-tumour immune response.

### Endothelial adhesion molecules regulate immune cell infiltration

For successful T cell extravasation, endothelial adhesion molecules (EAMs) such as intercellular adhesion molecule-1 (ICAM-1), ICAM-2, vascular endothelial cell adhesion molecule-1 (VCAM-1) and E-selectin are expressed on endothelial cells in response to inflammatory cytokines such as TNF-α, IFN-γ and IL-1 (65). However, exposure of vessel endothelium to pro-angiogenic factors such as VEGF and bFGF can repress the inflammatory cytokine-induced expression of EAMs (65). For example, the addition of VEGF and bFGF to endothelial cells *in vitro* inhibited IL-lα induced ICAM-1 upregulation by 15% and 33%, respectively (65).

Murine tumour models also demonstrated that endothelial cells exposed to bFGF and long-term VEGF display reduced ICAM-1 and VCAM-1 expression as well as decreased expression of TNF-α induced CXCL10 and CXCL11 T-cell chemoattractants (1, 66). Evidence has shown that VEGF interferes with the NF-kB pathway by degrading the IkB component, thus reducing the expression of TNF-α and its downstream molecules (66). This was confirmed ***via*** RT-PCR analysis which revealed that endothelial cells treated with VEGF elicited a 60-95% reduction in TNF-α expression (66). Moreover, renal cell carcinomas (RCC) can also interfere with the NF-kB pathway mediated expression of TNF-α by increasing P38-MAPK activity (66). This phenomenon termed ‘endothelial anergy’ causes an immunosuppressive reduction in immune cell infiltration, aiding tumour cell survival and inhibiting immunotherapeutic drug delivery (66, 67).

### Endothelial cells secreted extracellular vesicles in immunosuppression

Tumour endothelium-secreted extracellular vesicles (EVs) could induce reprogramming of immune cells. For instance, Lopatina et al., 2020 observed an increase in secretion of TGF-beta and IL10 by PBMC and to increase Treg expansion (68). These EVs have been shown to carry specific proteins (e.g. TGF-beta 1) and RNA (long non-coding RNA MALAT1) that are responsible for Treg differentiation and immunosuppression (68–70). Additionally, the authors reported that these EVs induced differentiation of monocytes to immunosuppressive macrophage type M2 (68). Interestingly, endothelial cell-secreted EVs containing miRNA-222 have been shown to induce downregulation of ICAM-1 on endothelial cell surface, which in turn reduces transmigration of immune cells (71). miR-10a from EVs derived from endothelial was shown to inhibit inflammatory signalling in monocytes through the targeting of several components of the NF-κB pathway, including IRAK4 (72). Thus, anti-tumour immunotherapies against EVs in combination with anti-angiogenic therapies may be an important therapeutic mechanism in the future.

### Abnormal ECM remodelling and immunosuppressive cells

Hypoxia-induced ECM remodelling in the TME alters the dynamic of physiological cellular and molecular crosstalk and processes such as migration and proliferation. In TME, ECM remodelling results in abnormal expression of various proteins such as collagen, fibronectin, versican, etc., that could result in immunosuppression. For instance, it has been shown that an excessive expression of versican, a chondroitin sulphate proteoglycan in the stroma of cervical cancer, was significantly associated with a low number of tumour infiltrating T cells, particularly CD8+ T cell, resulting in a reduced anti-tumour immune response (73). In contrast, TAMs, which share properties of M2 (immunosuppressive), are recruited in response to hypoxia *via* VEGFR1 activation, observed by a positive correlation between macrophage index and VEGF expression intensity in breast carcinoma samples (44). Furthermore, high M2 macrophage ratios have been associated with a poorer prognosis as shown in non-small lung cancer patients which displayed a 20.6% decrease in overall survival rate compared to their low M2 ratio counterparts (74), suggesting an anti-tumour immune phenotype in hypoxic TME. This abnormal ECM remodelling also results in an immature phenotype consisting of an unstable vessel wall with a discontinuous basement membrane and an irregular endothelial lining. This abnormal vascular architecture is excessively chaotic and leaky, creating, as described above, a hypoxic TME. This hypoxic condition dysregulates signalling that alters the expression of endothelial cell surface adhesion molecules as well as mediates the presence of several immunosuppressive immune cell types such as immature dendritic cells, TAMs, tumour-associated neutrophils and MDSCs, leading to a further reduction in anti-tumorigenic immune cell population within the TME, which has been extensively reviewed elsewhere (75).

However, the reduced immune cell population in the TME does not exclusively result from inhibited leukocyte extravasation but also increased leukocyte apoptosis. Human and mouse cancer models show that tumour endothelial cells display an increased expression of apoptotic Fas ligand in response to VEGF-A upregulation (76). Despite that endothelial Fas ligand can act selectively on CD8^+^ leukocytes, Treg cells display resistance due to their increased expression of c-FLIP which inhibits Fas ligand-induced apoptosis. This consequently prevents infiltration of cytotoxic leukocytes while maintaining the immunosuppressive Treg cell population within the TME (76). This increased immunosuppressive cell population generated by the tumour vasculature includes TAMs, Tregs and MDSCs which aid the immunosuppressive profile of the TME (77–81). For example, pro-tumorigenic polymorphonuclear neutrophil myeloid-derived suppressor cells (PMN-MDSCs) can also suppress CD8^+^ leukocytes and induce pro-angiogenic MMP9 release (82). Furthermore, other pro-tumorigenic neutrophils such as N2 tumour-associated neutrophils been linked to poor prognosis and cancer progression but the extent of whether this association is causative or correlative remains unclear (82).

## Regulating genes of the tumour vasculature

Dysregulations of the TME contributing to the angiogenic switch have been linked to distinct mechanisms including anomalous expression levels of HIF-1, pro-angiogenic factors VEGF and bFGF as well as anti-angiogenic factors such as thrombospondin-1 (TSP-1) (83, 84). The tumour suppressor gene *p53* regulates these factors and mediates cellular functions such as apoptosis, DNA repair, cell cycle development and angiogenesis inhibition (83). However, *p53* is commonly mutated in cancers, thereby promoting pathological angiogenesis (83).

*P53* knockout human colon adenocarcinoma cells displayed increased HIF-1α expression in comparison to *p53* homozygous cells under hypoxic conditions (1% O2) (85). Regardless, no significant difference in p53 mRNA expression was detected, posing further investigations into the mechanistic pathway. These results indicated that p53 loss-of-function mutations prevent the functional ubiquitination and degradation of HIF-1α(85). In addition, p53 demonstrated a possible role in controlling HIF-1β expression *via* regulating microRNA transcription (86). RNA analysis has shown the presence of a p53 binding site 1,811bp upstream of the pantothenate kinase 1 intron which contains the encoding section for microRNA-107. Upon p53 binding, microRNA-107 levels increased and subsequently inhibited HIF-1β expression (86). Therefore, p53 mutations may lead to both increased HIF-1α and HIF-1β expression, thereby promoting angiogenesis by upregulating factors such as VEGF (86). Furthermore, p53 mutations have also been demonstrated to upregulate VEGF expression in human colon, bladder, and breast cancer surgical specimens as p53 acts directly on the VEGF promoter region (44, 84).

Additionally, the anti-angiogenic ECM glycoprotein TSP-1 is implicated in reducing cancer cell proliferation, survival as well as motility and is upregulated by p53 binding to the encoding THBS1 promoter region (84, 87, 88). This suggests that p53 mutations downregulating TSP-1 may further contribute to the formation of abnormal tumour vasculature (84, 88).

Contrary to tumour suppressor genes, oncogenes can enhance angiogenesis when their activity is upregulated *via* gain-of-function mutations. For example, *PTPN11* encodes the tyrosine phosphate SHP2, which is often overactivated in tumour endothelial cells (89). Knockout or inhibition of *Shp2* in human umbilical vein endothelial cells (HUVECs) demonstrated reduced SOX7 and SOX18 transcription factor expression, thus inhibiting cell motility, proliferation, and tubular vessel formation (89). This was supported by data comparing relative SOX7 levels in *Shp2*-knockout (Shp2^KO^) and *Shp2* homozygous HUVECs which showed a significant reduction in Shp2^KO^ HUVECs (89). Both transcription factors have similar roles and re-expressing SOX7 in Shp2^KO^ HUVECs restored cellular functions while also increasing vessel branching and density (89). Furthermore, inducing SOX7 expression in Shp2^KO^ mice decreased basement membrane and pericyte coverage, indicated by results showing that Shp2^KO^ mice displayed a relative branching index reduction of 0.54 and a 0.58 (arbitrary units) increase in relative pericyte coverage when compared to *Shp2* homozygous mice (89). These were quantitatively measured using computer image analysis. Additionally, Shp2^KO^ endothelial cells displayed increased PDGF-BB expression, which aids vessel maturation and thus may be implicated in the observed disparity in pericyte and basement membrane coverage of Shp2^KO^ mice (89). Finally, tumour mouse models lacking *Sox7* expression within endothelial cells demonstrated reduced tumour growth, Treg penetration, endothelial VEGFR2 expression and aberrant vessel morphology (90). Evidently, SH2 and SOX7 pose as ideal targets to induce vascular normalisation (89, 90).

## Therapeutics targeting the tumour vasculature

### Vascular normalisation

Many treatments seek to improve the tumour vasculature by simultaneously removing aberrant vessels and stabilising those that are functional. This aims to restore the vasculature to a normal physiological state, *via* a process termed ‘vascular normalisation’ (28). Currently available anti-angiogenic drugs can be broadly divided into three groups. These are receptor tyrosine kinase (RTKs) inhibitors, anti-VEGF or anti-RTK antibodies and downstream angiogenic signalling inhibitors (91). Although monotherapeutic use of these drugs has displayed variable effectiveness and reached to FDA approval only in some specific tumour types (92), addition of these anti-angiogenic agents to standard chemotherapy or radiotherapy has shown promise in improving their efficacy (4, 6). The inconsistency in efficacy has been associated with the varying innate drug susceptibility of certain tumours and the development of acquired drug resistance after treatment (92). There are currently many FDA approved angiogenesis inhibitors used in cancer treatment targeting proangiogenic molecules, with the vast majority targeting VEGFR (93). These treatments include bevacizumab and ramucirumab, which target VEGF-A and VEGFR2, respectively and RTK inhibitors sunitinib, axitinib, sorafenib and lenvatinib (93). Furthermore, the combinational use of anti-angiogenic drugs and immunotherapy [reviewed elsewhere (94, 95)] has been demonstrated to increase the efficacy of the latter, making it a promising target to replace cytotoxic treatments that often accompany quality of life reducing side effects such as alopecia, mucositis, and cardiotoxicity (4, 96). Importantly, these anti-angiogenic drugs continue to be utilised in clinical trials to explore their effects on cancer therapy. Some of these ongoing clinical trials are shown in **Table 1**.

**Table 1:** Clinical Trials Targeting Cancers and Tumours using Anti-angiogenic Treatment.

Drug/Treatment	Target	Phase	Application	(Estimated)Start Date -Predicted End Date	NCT Number/Source
**SIBP04** + Paclitaxel + Carboplatin OR **Avastin** + Paclitaxel + Carboplatin	VEGF	Phase 3	Non-squamous Non-small-cell Lung Cancer	Apr. 17, 2020 – Sep. 30, 2022	NCT05318443
**Bevacizumab** + Atezolizumab + Gemcitabine + Cisplatin OR Gemcitabine + Cisplatin	VEGF	Phase 2	Combined hepatocellular carcinoma and Cholangiocarcinoma	Feb. 11, 2022 – Jan. 31, 2025	NCT05211323
**Bevacizumab** + STRO-002	VEGF	Phase 1	Ovarian Cancer	Mar. 22, 2022 – Jan. 2024	NCT05200364
**Apatinib** + Albumin-Bound Paclitaxel OR **Bevacizumab** + Albumin-Bound Paclitaxel OR Albumin-Bound Paclitaxel	Anti-angiogenic (VEGFR2, VEGF)	Phase 2	Triple-negative Breast Cancer	Jan. 14, 2022 – Jun. 1, 2024	NCT05192798
**(Bevacizumab** +) Chemotherapy OR **Bevacizumab** + Atezolizumab (+ Chemotherapy) OR Cetuximab + Chemotherapy Chemotherapy = Cisplatin/Carboplatin + Docetaxel	Anti-angiogenic (VEGF)	Phase 2/3	Metastatic/Advanced Head and Neck Cancer	Dec. 16, 2021 – Dec. 15, 2027	NCT05063552
**Bevacizumab** + Ensartinib Carboplatin + Pemetrexed	VEGF	Phase 1	ALK-Positive Lung Non-Small Cell Carcinoma	Mar. 18, 2021 – Sep. 23, 2022	NCT04837716
**Bevacizumab** + Riluzole + mFOLFOX6	VEGF	Phase 1	Metastatic Colorectal Cancer	Apr. 2, 2021 – Dec. 31, 2024	NCT04761614
**Bevacizumab** + Irinotecan sucrosofate	VEGF	Phase 2	Ovarian, Fallopian tube, Primary Peritoneal Cancer	Mar. 16, 2021 – July. 1, 2024	NCT04753216
**Bevacizumab** + Atezolizumab	VEGF	Phase 2	Resectable Liver Cancer	Feb. 10, 2021 – Dec. 31, 2027	NCT04721132
**Avastin** + Placebo + XELOX OR **HLX04** + HLX10 + XELOX	VEGF	Phase 2/3	Metastatic Colorectal Cancer	Mar. 10, 2021 – June. 30, 2025	NCT04547166
**Bevacizumab** + Brigatinib	VEGF	Phase 1	ALK-Rearranged Non-Small Cell Lung Cancer	Mar. 9, 2020 – Nov. 1, 2023	NCT04227028
**Bevacizumab** + Osimertinib OR Osimertinib	VEGF	Phase 3	Non-Small Cell Lung Cancer	Oct. 22, 2020 – Sep. 1, 2025	NCT04181060
**Bevacizumab** + Dasatinib + mFOLFOX	VEGF	Phase 1	Gastrointestinal Cancer	Sep. 2, 2020 – Dec. 31, 2022	NCT04164069
**Bevacizumab** + Irinotecan + TAS-102	VEGF	Phase 2	Metastatic/Unresectable Colorectal Cancer	Dec. 27, 2019 – Apr. 22, 2024	NCT04109924
**Bevacizumab**/Nab-Paclitaxel + IPI-549 + Atezolizumab	VEGF	Phase 2	Triple-Negative Breast Cancer and Renal Cell Cancer	Dec. 17, 2019 – Aug. 1, 2022	NCT03961698

Table listing most recent active + not recruiting, recruiting, and enrolling clinical trials from clinicaltrials.gov (refer via NCT number) targeting cancers/tumours that employ the use of anti-angiogenic drugs that target VEGF. Clinical trials included were researched on the 21^st^ July 2022.

Anti-angiogenic drugs are listed in **bold**.

### Anti-VEGF antibody therapy - bevacizumab

Bevacizumab is an anti-VEGF IgG1 humanised monoclonal antibody, and the first anti-angiogenic drug approved for colorectal cancer treatment by the Food and Drug Administration (FDA) (4, 97). At the base of the angiogenic switch, VEGF acts as a highly potent pro-angiogenic factor, promoting endothelial cell proliferation and migration (98). However, bevacizumab use has been demonstrated to prevent VEGF from binding to its receptors, in particular VEGFR1 and VEGFR2, which activate multiple angiogenic pathways (99). For example, neuroblastoma xenograft models demonstrated that bevacizumab induced vessel regression, observed by a 75% decrease in vessel length 7 days after administration (100). This consequently improved tumour vascular architecture and structure (100). Additionally, 7 days after bevacizumab treatment, tumours displayed a 70% reduction in microvessel density, 60% reduction in tumour interstitial pressure and 48% reduction in blue dye extravasation representing reduced vessel permeability, thereby improving overall tumour perfusion (100). Besides its anti-angiogenic effects, bevacizumab also exhibits cytotoxic action in chronic lymphocyte leukaemia (CCL) by upregulating pro-apoptotic Bad, Bax, and Akt proteins while downregulating anti-apoptotic Mcl-1 protein (99). This protein expression profile increases caspase 3 and caspase 9 activity, triggering CCL apoptosis (99).

Bevacizumab is often used to increase chemotherapy efficacy, which led to its FDA approval (100). Demonstrated in metastatic colorectal cancer patients, concurrent treatment of bevacizumab and irinotecan, fluorouracil, and leucovorin (IFL) chemotherapy significantly increased median survival duration by 30.1% compared to patients treated with IFL and placebo (97). Furthermore, median progression-free survival duration increased by 71.0% from 6.2 to 10.6 months with bevacizumab and IFL treatment compared to placebo and IFL treatment (97). Xenograft tumour models further demonstrated bevacizumab’s ability to augment the efficacy of chemotherapy (100). Combinational and delayed administration of topotecan to bevacizumab-treated mice elicited enhanced tumour volume reduction compared to their monotherapeutically topotecan or bevacizumab treated counterparts (100). These results were consistent with data showing that delayed chemotherapy administration to bevacizumab-treated mice improved intratumoral chemotherapeutic drug penetration as 3 days after bevacizumab treatment, topotecan tumour penetration increased by 22% compared to size-matched controls (100). Furthermore, combining bevacizumab with various chemotherapies (paclitaxel and ixabepilone) in patients with *TP53* mutations improved PFS and OS compared to chemotherapy alone (101). However, cediranib, an anti-angiogenic tyrosine kinase inhibitor, demonstrated higher cell cycle abrogation and synergy with chemotherapy compared to bevacizumab in endometrial cancer models *in vitro* (102).

### Receptor tyrosine kinase inhibitor therapy - sunitinib

Sunitinib is an FDA approved multi-RTK inhibitor used to treat RCCs, imatinib-resistant gastrointestinal stromal tumours (GIST) and advanced pancreatic neuroendocrine tumours (PNETs) (103, 104). VEGF and PDGF receptors are commonly overexpressed on tumour vessel pericytes and endothelial cells, causing increased angiogenesis. Since sunitinib can prevent ligand binding to VEGFRs, PDGFRs, c-Kit, Flt3 and RET kinases, it inhibits their activation and subsequent angiogenic effects mediated by them (105–107).

*In vitro* experiments using human lung microvascular endothelial cells (HLMECs) showed sunitinib treatment reduced endothelial cell proliferation and invasion due to sunitinib-mediated VEGFR2 inhibition, while *in vivo* experiments using RCC xenograft, mice presented a significant reduction in microvessel density, culminating in tumour growth inhibition (107). Furthermore, sunitinib-treated prostate cancer xenograft models demonstrated an average 0.15 reduction in nitroimidazole standardized uptake value (uptake based on radioactivity administered, radioactivity detected in volume of interest and body weight) measured *via* microPET analysis (108, 109). This correlated to decreased HIF-1α expression and thus indicates reduced tumour hypoxia (108). This was stipulated to result from sunitinib inhibiting VEGFR and PDGFR, preventing Akt and Erk1/2 signalling pathways that induce HIF-1α expression (108). As hypoxia is a known inhibitor of radiotherapy, subsequent investigations using *in vivo* mouse models demonstrated that sunitinib administration increased prostate cancer cell susceptibility to irradiation-mediated apoptosis (108).

Unfortunately, sunitinib has demonstrated variable results in its combinational efficacy with chemotherapy. The notable phase 2 clinical trial employing both sunitinib and gemcitabine to treat RCC, displayed an improved objective response rate (ORR) compared to monotherapies of either drug (110). However, other studies, such as those covering metastatic breast cancer, revealed no statistical significance in progression-free survival or response rate when sunitinib was excluded or used in combination with capecitabine therapy (111). Furthermore, the combination of both drugs increased toxicity risk in patients, which remains a current challenge in implementing sunitinib therapy (111, 112).

Interestingly, trials employing pre-treatment of sunitinib before chemotherapy in breast cancer patients, demonstrated a statistically significant 12.46% increase in vascular normalisation index (VNI) compared to pre-treatment with bevacizumab (113). The VNI biomarker is defined by vessel permeability, volume and collagen IV plasma levels (relative to basement membrane thinning) which allows for quantification of vascular normalisation (114, 115). The observed VNI therefore indicates anti-angiogenic pre-treatment may improve chemotherapeutic drug delivery, particularly in cancers where concurrent administration has not proved effective (113).

Besides sunitinib’s anti-angiogenic effects, sunitinib has also been shown to improve the immunosuppressive lymphocyte profile in the TME (116). In hepatocellular carcinoma mouse models, flow cytometry analysis revealed that tumour-bearing mice displayed an increased population of Treg cells compared to tumour-free controls (116). These Treg cells also presented with upregulated immunosuppressive IL-10 and TGF-β cytokine production, consequently inhibiting CD8^+^ T cell immune responses (116). In the presence of Tregs isolated from tumour-bearing mice, stimulated tumour-antigen-specific (TAS) CD8^+^ T cells exhibited reduced cell proliferation and IFN-γ production when compared to CD8^+^ T cells stimulated with Tregs isolated from tumour-free mice (116). Despite this, when TAS CD8^+^ T cells were stimulated with Tregs from tumour-bearing mice treated with sunitinib, T cell proliferation and IFN-γ production was restored (116). This association was supported as sunitinib-treated tumour-bearing mice exhibited a significantly reduced Treg population in spleen, draining-lymph node, and liver samples compared to tumour-bearing controls (116). Crucially, IL-10 and TGF-β cytokine production was also restored to levels comparable to Tregs from tumour-free control mice, improving the CD8^+^ T cell-mediated cytotoxic response (116).

### Investigatory receptor tyrosine kinase inhibitors in clinical trials

There are some investigatory RTK inhibitors that are not approved by FDA. Some of these agents include cediranib, motesanib and surufatinib which inhibit VEGFR isoforms. Cediranib and motesanib also inhibit platelet-derived growth factor receptors and cediranib and surufatinib additionally inhibit fibroblast growth factor receptor (FGFR1).

Inhibition of multiple RTKs is an advantageous feature in cancer treatment. A study by Bi et al., 2021 has shown that cediranib combination with paclitaxel had higher cell death while bevacizumab combination with paclitaxel treatment showing a small insignificant change in cell death in endometrial cancer cells (102). In phase III trials, cediranib had limited benefit in metastatic colorectal cancer (NCT00384176), non-small cell lung cancer (NCT00795340), and recurrent glioblastoma (NCT00777153) (117–119). In platinum-sensitive ovarian cancer (NCT00532194) cediranib combination with chemotherapy then cediranib maintenance therapy had a significant improvement in progression-free survival when compared to chemotherapy (median, 11 months vs 8.7 months) but it was also associated with greater toxicity (120). After a follow-up, median survival with cediranib combination with chemotherapy then cediranib maintenance therapy was higher by 7.4 months when compared to chemotherapy (27.3 months vs 19.9 months) (121). On the other hand, in platinum-sensitive ovarian cancer, comparison between cediranib combination with olaparib to chemotherapy had no significant improvement in progression-free survival (median, 10.4 vs 10.3 months) (122).

Motesanib and surufatinib are reviewed in Qin et al., 2019 (103). Furthermore, there is a need for further research to clarify the role of these newer RTK inhibitors in cancer treatment. Additionally, other agents such as fruquintinib (NCT02314819), nintedanib (NCT00805194), and anlotinib (NCT02388919) have obtained regulatory approvals other than FDA could be also potential agents requiring further research for defining their clinical efficacy in various cancer treatment with drug combinations.

## Challenges and resistance to anti-angiogenic therapy

Despite that anti-angiogenic therapies have shown success in certain tumours, many cancers including breast, pancreatic and prostate cancers initially display or develop resistance after treatment (92). As demonstrated by bevacizumab and sunitinib, a large proportion of anti-angiogenic therapies target VEGF/VEGFR pathways, which are not exhaustive mediators of angiogenesis. Many other growth factors such as Ang-2, FGF, PDGF and TGF-β continue to support angiogenesis in cancers which display anti-VEGF resistance (123). Additionally, tumours can employ non-angiogenic mechanisms to obtain blood supply, thereby rendering anti-angiogenic therapy as ineffective. This was observed in sorafenib-treated hepatocellular carcinoma mouse models, which displayed drug resistance 38 days after initial treatment, identified by an increase in human choriogonadotropin rate of 28.3 mIU/mg/day that represents an increase in invasive tumour growth rate (24). Later histological analysis revealed sorafenib early-resistant tumours displayed a 51.7% increase in dependence on co-opted blood vessel supply compared to control tumours (24). Drug-resistant tumours can also obtain blood supply *via* vasculogenic mimicry as displayed in RCC cell lines and xenograft mouse models (124). Tumours from sunitinib-treated RCC mice displayed characteristic VM biomarker alterations, including increased PAS staining and reduced CD31 expression when compared to control mice (124). Observed RCC tumour progression during sunitinib treatment has been linked to VM induction, possibly *via* sunitinib upregulating ERβ that causes HIF-2α production (124, 125).

Even though anti-angiogenic therapies have shown success, the transient time that vascular normalisation is elicited after treatment, termed the ‘vascular normalisation window’ poses a major challenge (3). Maximising this period is essential to improving chemotherapy and radiotherapy effectiveness, but it is difficult to monitor due to a lack of biomarkers and effective parameters needed for optimal dosing strategies (3, 62, 126). However, techniques such as blood-oxygen-level-dependent MRI and dynamic contrast-enhanced MRI can be used to non-invasively determine tumour hypoxia, vessel perfusion and vessel permeability (62, 127). The oxymoronic effects of anti-angiogenic therapy have also been described to cause undesirable vessel regression leading to hypoxia (128). Hypoxia mediated HIF-1α expression, upregulates factors that can recruit immunosuppressive bone marrow-derived cells (BMDCs), causing a range of downstream effects. For example, these cells can express MMP9 to promote ECM degradation and increase VEGF bioavailability which supports angiogenesis (128).

## Future perspectives – novel nanoparticle therapy

The discussed limitations of anti-angiogenic therapy are difficult to overcome, evident by drug resistance and toxicity observed in clinical trials (129). Notably, the aberrant perfusion and architecture of tumour vessels limits the delivery of drugs to the relevant site, and the normalisation window consistently hurdles effective dosing that also prevents excess vessel regression (126, 129). Nanoparticles can potentially bypass these obstacles *via* specific tissue targeting and improving drug pharmacokinetics by reducing dosage and enhancing drug stability (9). Furthermore, therapeutic nanoparticles are highly diverse, consisting of inorganic, lipid and polymer formulations whose varying characteristics can be utilised (9).

Liposomal nanoparticles have shown some promise when combined with doxorubicin. Doxorubicin chemotherapy is often utilised in the treatment of breast cancer but cumulative dosing poses challenges in preventing toxicity induced cardiomyopathy (130). Evidence has shown that liposomal-doxorubicin formulations induced 64% fewer cases of toxicity induced congestive heart failure compared to conventional doxorubicin in a metastatic breast cancer clinical trial. Despite the decrease in cardiotoxicity, no statistically significant difference in patient survival time was observed (130). Moreover, improvements upon liposomal nanoparticles *via* pegylation can allow for prolonged systemic circulation time. Pegylated liposomal-doxorubicin, known as Doxil/Caelyx achieves this by surface coating the liposome-encapsulated drug with methoxy-poly-ethylene-glycol molecules, permitting immune evasion from the reticuloendothelial system (131).

Using nanoparticle technology to improve drug delivery in solid tumours has largely been aimed at taking advantage of the enhanced permeability and retention (EPR) effect elicited by the tumour vasculature (132). The hyperpermeability of tumour blood vessels is proposed to result from attenuated endothelial junctions and the presence of vesiculo-vacuolar organelles (VVOs) in microvessels that can allow transendothelial extravasation (133, 134). The EPR effect attributes this to increasing the extravasation of nanoparticles in tumour vessels, thus increasing drug accumulation in tumours while reducing drug accumulation in healthy tissues (132). However, the effect appears more prevalent in xenograft mouse models than in human tumours, leading to scepticism regarding its impact in clinical use (132).

Despite this, nanoparticle-mediated technology can be effective in cancer therapy by inducing other mechanisms including vascular normalisation (135). Gold nanoparticles (AuNPs) contain a gold core surrounded by a monolayer that can contain specialised ligands to improve delivery to target cells (136). For example, AuNPs were conjugated to folic acid ligands to improve their targeting to tumours as folate receptor overexpression is observed in many cancers (135). Furthermore, the structure of AuNPs is variable depending on their size, which could be used to improve their efficacy (136). For example, AuNPs have demonstrated the ability to reduce chaotic vessel architecture, permeability and hypoxia while increasing pericyte coverage and T cell infiltration (135, 137). These effects were associated with AuNP treatment upregulating the expression of the semaphorin 3A cytokine (SEMA3A) in gastric adenocarcinoma cells which can subsequently inhibit the TGF-β mediated SMAD2/3 signalling pathway in endothelial cells (135). Lastly, AuNP treatment has also been shown to downregulate the expression of pro-angiogenic VEGF-A in gastric adenocarcinoma cells, attenuating angiogenesis (135).

As previously discussed, cathepsin D and L play potent proangiogenic roles within the tumour microenvironment. In an attempt to inhibit this potent proangiogenic role of cathepsins D and L in the extracellular space, we tested effects of highly compatible graphene oxide (GO) *in vitro* (138). Our group showed for the first time that GO could be used as a strong inhibitor for cathepsins D and L in a time, dose and pH dependent manner. Using analytical tools such as Raman scattering system, Fourier Transform Infrared Spectroscopy (FTIR), water contact angles and surface energy, we demonstrated a strong bonding between the enzymes GO and an adsorption capacity of GO which resulted in denaturation of the enzymes functional active sites. The cationic and hydrophilic residues on the surface of GO mediates adsorption of the enzymes, resulting in denaturation and deactivation. The mechanistic aspects of protein adsorption and/or protein corona formation as a result of the interaction of proteins with graphene may involve electrostatic and hydrophobic interactions (139). However, further studies are required to examine this promising anti-metastatic effect of GO in cell culture and *in vivo* models.

Recent developments in nanoparticle-based formulations have harnessed huge attention in detecting and treating cancer. The integration and conjugation of nanoparticles with wide-ranging therapeutic agents or ‘classical’ chemotherapeutic drugs provides innovative approaches to release or activate (in response to external or internal sources such as light, ultrasound, pH) the therapeutic agent in a controlled, safe and targeted manner. Nanoparticles-based approaches offer several advantages over conventional delivery or treatment modalities such as enhanced delivery of nanoparticles due to EPR, improved pharmacokinetics, precise control over release mechanisms, surface modification with specific cellular or sub-cellular targeting ligands, tunability of size, shape, morphology and surface charge as well as the ease of functionalisation with biomolecules (e.g., RNA, antibodies, nanobodies and other payloads). Another advantage is the two-in-one function of nanoparticles where they can be used as a theranostics agent, a combination of diagnostics and treatment. Over recent years, more than 15 nanoparticles have clinically been approved for diagnostics and therapeutic purposes (140, 141). In comparison to conventional treatment options, nanoparticles offer unique physiochemical features which have the ability to deliver drugs to the key players of the TME. When nanoparticles interact with different components of the TME, they also have the ability to modify the immunosuppressive environment. For instance, their interactions with blood vessels can induce hypoxia. Nanoparticles encapsulated with immunosuppressive factors such as VEGF and TGF-β can release these factors in a sustained fashion thereby leading to an abnormal tissue dynamic (142, 143). Such critical roles of nanoparticles in the TME are beyond the scope of the article and we refer the reader to specialised literature on this (144, 145).

Furthermore, the TME plays a crucial role in the biodistribution and biological fate of nanoparticles, although more in-depth investigations are required to explore the role of the TME in targeting nanoparticles towards the site of action. In this review we mainly summarise the crosstalk between the immune element of the TME and local tumour vascular endothelial cells with a brief discussion on the role of emerging nanoparticles. Nanoparticles have the potential to affect the abnormal roles of the TME, which in turn can also minimise the burden of drug resistance development thereby significantly improving the therapeutic effects in a targeted fashion.

## Conclusion

The tumour vasculature’s features and their complex interactions with the TME are established to perpetuate angiogenesis and suppress the immune response. Associated mechanisms include upregulating pro-angiogenic factor expression, reducing therapeutic drug delivery and repressing effector T cell infiltration into the tumour parenchyma. These barriers consequently reduce chemotherapy, radiotherapy, and immunotherapy success in solid tumours, emphasising tumour vasculature formation as an important therapeutic target. Currently, anti-angiogenic treatments that promote vascular normalisation are used, however, their efficacy has proved inconsistent in clinical trials, with many tumours displaying initial and acquired drug resistance. The ambiguous processes inducing resistance remain to be clarified but include tumours exploiting non-VEGF pro-angiogenic pathways, obtaining blood supply *via* non-angiogenic methods such as vasculogenic mimicry and ineffective utilisation of the normalisation window during treatment. Therefore, exploring alternative therapies combatting these obstacles remains essential to improving cancer treatment.

Progress has been made in this field, including nanoparticle therapy, however, improving the understanding of inducing vascular normalisation will allow treatments to further leverage the normalisation window and thus reduce the risk of developing drug resistance. Furthermore, many anti-angiogenic treatments continue to predominantly focus on targeting VEGF however developing other therapies that target additional pro-angiogenic pathways may allow for the successful treatment of a wider variety of tumours. Lastly, the importance of non-angiogenic mechanisms in acquiring tumour blood supply remains to be fully explored so that therapies targeting these mechanisms can be developed. Evidently, furthering the understanding of the tumour vasculature’s effect on angiogenesis, immunosuppression and treatment could vastly improve the field of cancer therapy.

## Author contributions

ZI, TT, JY and MP drafted the manuscript. DZ and JY reviewed and edited the manuscript. NA contributed with discussions and critical revision of the manuscript. MP edited the manuscript and conceptualised, supervised and administered the project. All authors contributed to the article and approved the submitted version.

## Funding

This work was supported by funding Vetenskaps-rådet (The Swedish Research Council, 2017-04663) and Carl Tryggers Stiftelse (CTS:18:279).

## Conflict of interest

The authors declare that the research was conducted in the absence of any commercial or financial relationships that could be construed as a potential conflict of interest.

## Publisher’s note

All claims expressed in this article are solely those of the authors and do not necessarily represent those of their affiliated organizations, or those of the publisher, the editors and the reviewers. Any product that may be evaluated in this article, or claim that may be made by its manufacturer, is not guaranteed or endorsed by the publisher.

## References

[1] Dirkx AE Oude Egbrink MG Kuijpers MJ van der Niet ST Heijnen VV Bouma-ter Steege JC. Tumor angiogenesis modulates leukocyte-vessel wall interactions *in vivo* by reducing endothelial adhesion molecule expression. Cancer Res (2003) 63(9):2322–9. 12727857

[2] Kuehlbach C Hensler S Mueller MM. Recapitulating the angiogenic switch in a hydrogel-based 3D *In vitro* tumor-stroma model. Bioeng (Basel) (2021) 8(11). doi: 10.3390/bioengineering8110186 PMC8614676 34821752

[3] Zhao Y Yu X Li J. Manipulation of immunevascular crosstalk: new strategies towards cancer treatment. Acta Pharm Sin B (2020) 10(11):2018–36. doi: 10.1016/j.apsb.2020.09.014 PMC7714955 33304777

[4] Yang T Xiao H Liu X Wang Z Zhang Q Wei N. Vascular normalization: A new window opened for cancer therapies. Front Oncol (2021) 11:719836. doi: 10.3389/fonc.2021.719836 34476218 PMC8406857

[5] Hashizume H Baluk P Morikawa S McLean JW Thurston G Roberge S. Openings between defective endothelial cells explain tumor vessel leakiness. Am J Pathol (2000) 156(4):1363–80. doi: 10.1016/S0002-9440(10)65006-7 PMC1876882 10751361

[6] Fukurnura D Kloepper J Amoozgar Z Duda DG Jain RK. Enhancing cancer immunotherapy using antiangiogenics: opportunities and challenges. Nat Rev Clin Oncol (2018) 15(5):325–40. doi: 10.1038/nrclinonc.2018.29 PMC5921900 29508855

[7] Martin JD Seano G Jain RK. Normalizing function of tumor vessels: Progress, opportunities, and challenges. Annu Rev Physiol (2019) 81:505–34. doi: 10.1146/annurev-physiol-020518-114700 PMC6571025 30742782

[8] Graham K Unger E. Overcoming tumor hypoxia as a barrier to radiotherapy, chemotherapy and immunotherapy in cancer treatment. Int J Nanomed (2018) 13:6049–58. doi: 10.2147/IJN.S140462 PMC6177375 30323592

[9] Mattheolabakis G Mikelis CM. Nanoparticle delivery and tumor vascular normalization: The chicken or the egg? Front Oncol (2019) 9:1227. doi: 10.3389/fonc.2019.01227 31799190 PMC6863425

[10] Sakurai Y Abe N Yoshikawa K Oyama R Ogasawara S Murata T. Targeted delivery of lipid nanoparticle to lymphatic endothelial cells *via* anti-podoplanin antibody. J Control Release (2022) 349:379–87. doi: 10.1016/j.jconrel.2022.06.052 35787913

[11] Mander KA Finnie JW. Tumour angiogenesis, anti-angiogenic therapy and chemotherapeutic resistance. Aust Vet J (2018) 96(10):371–8. doi: 10.1111/avj.12747 30255577

[12] Chang SH Kanasaki K Gocheva V Blum G Harper J Moses MA. VEGF-a induces angiogenesis by perturbing the cathepsin-cysteine protease inhibitor balance in venules, causing basement membrane degradation and mother vessel formation. Cancer Res (2009) 69(10):4537–44. doi: 10.1158/0008-5472.CAN-08-4539 PMC2683911 19435903

[13] Gerhardt H Golding M Fruttiger M Ruhrberg C Lundkvist A Abramsson A. VEGF guides angiogenic sprouting utilizing endothelial tip cell filopodia. J Cell Biol (2003) 161(6):1163–77. doi: 10.1083/jcb.200302047 PMC2172999 12810700

[14] Zahra FT Choleva E Sajib MS Papadimitriou E Mikelis CM. *In vitro* spheroid sprouting assay of angiogenesis. Methods Mol Biol (2019) 1952:211–8. doi: 10.1007/978-1-4939-9133-4_17 30825177

[15] Yetkin-Arile B Vogels IMC Neyazi N van Duinen V Houtkoopers RH van Noorden CJF. Endothelial tip cells in vitro are less glycolytic and have a more "exible response to metabolic stress than non-tip cells. Sci Rep (2019) 9. doi: 10.1038/s41598-019-46503-2 PMC6639367 31320669

[16] Viallard C Larrivee B. Tumor angiogenesis and vascular normalization: alternative therapeutic targets. Angiogenesis (2017) 20(4):409–26. doi: 10.1007/s10456-017-9562-9 28660302

[17] Sun B Qie S Zhang S Sun T Zhao X Gao S. Role and mechanism of vasculogenic mimicry in gastrointestinal stromal tumors. Hum Pathol (2008) 39(3):444–51. doi: 10.1016/j.humpath.2007.07.018 18261629

[18] Wei X Chen Y Jiang X Peng M Liu Y Mo Y. Mechanisms of vasculogenic mimicry in hypoxic tumor microenvironments. Mol Cancer (2021) 20(1):7. doi: 10.1186/s12943-020-01288-1 33397409 PMC7784348

[19] Angara K Rashid MH Shankar A Ara R Iskander A Borin TF. Vascular mimicry in glioblastoma following anti-angiogenic and anti-20-HETE therapies. Histol Histopathol (2017) 32(9):917–28. doi: 10.14670/HH-11-856 PMC5476524 27990624

[20] Sun H Zhang D Yao Z Lin X Liu J Gu Q. Anti-angiogenic treatment promotes triple-negative breast cancer invasion *via* vasculogenic mimicry. Cancer Biol Ther (2017) 18(4):205–13. doi: 10.1080/15384047.2017.1294288 PMC5450737 28278077

[21] Latacz E Caspani E Barnhill R Lugassy C Verhoef C Grunhagen D. Pathological features of vessel co-option versus sprouting angiogenesis. Angiogenesis (2020) 23(1):43–54. doi: 10.1007/s10456-019-09690-0 31655928

[22] Zhang Y Wang S Dudley AC. Models and molecular mechanisms of blood vessel co-option by cancer cells. Angiogenesis (2020) 23(1):17–25. doi: 10.1007/s10456-019-09684-y 31628560 PMC7018564

[23] Frentzas S Simoneau E Bridgeman VL Vermeulen PB Foo S Kostaras E. Vessel co-option mediates resistance to anti-angiogenic therapy in liver metastases. Nat Med (2016) 22(11):1294–302. doi: 10.1038/nm.4197 PMC5104270 27748747

[24] Kuczynski EA Yin M Bar-Zion A Lee CR Butz H Man S. Co-Option of liver vessels and not sprouting angiogenesis drives acquired sorafenib resistance in hepatocellular carcinoma. J Natl Cancer Inst (2016) 108(8). doi: 10.1093/jnci/djw030 PMC5017954 27059374

[25] Bridgeman VL Vermeulen PB Foo S Bilecz A Daley F Kostaras E. Vessel co-option is common in human lung metastases and mediates resistance to anti-angiogenic therapy in preclinical lung metastasis models. J Pathol (2017) 241(3):362–74. doi: 10.1002/path.4845 PMC5248628 27859259

[26] Abramsson A Berlin O Papayan H Paulin D Shani M Betsholtz C. Analysis of mural cell recruitment to tumor vessels. Circulation (2002) 105(1):112–7. doi: 10.1161/hc0102.101437 11772885

[27] Padera TP Stoll BR Tooredman JB Capen D di Tomaso E Jain RK. Pathology: cancer cells compress intratumour vessels. Nature (2004) 427(6976):695. doi: 10.1038/427695a 14973470

[28] Magnussen AL Mills IG. Vascular normalisation as the stepping stone into tumour microenvironment transformation. Brit J Cancer (2021) 125(3):324–36. doi: 10.1038/s41416-021-01330-z PMC8329166 33828258

[29] Yamakawa M Liu LX Date T Belanger AJ Vincent KA Akita GY. Hypoxia-inducible factor-1 mediates activation of cultured vascular endothelial cells by inducing multiple angiogenic factors. Circ Res (2003) 93(7):664–73. doi: 10.1161/01.RES.0000093984.48643.D7 12958144

[30] Rodriguez D Watts D Gaete D Sormendi S Wielockx B. Hypoxia pathway proteins and their impact on the blood vasculature. Int J Mol Sci (2021) 22(17). doi: 10.3390/ijms22179191 PMC8431527 34502102

[31] Hendriksen EM Span PN Schuuring J Peters JP Sweep FC van der Kogel AJ. Angiogenesis, hypoxia and VEGF expression during tumour growth in a human xenograft tumour model. Microvasc Res (2009) 77(2):96–103. doi: 10.1016/j.mvr.2008.11.002 19118564

[32] Moon WS Rhyu KH Kang MJ Lee DG Yu HC Yeum JH. Overexpression of VEGF and angiopoietin 2: a key to high vascularity of hepatocellular carcinoma? Mod Pathol (2003) 16(6):552–7. doi: 10.1097/01.MP.0000071841.17900.69 12808060

[33] Yoshiji H Kuriyama S Noguchi R Yoshii J Ikenaka Y Yanase K. Angiopoietin 2 displays a vascular endothelial growth factor dependent synergistic effect in hepatocellular carcinoma development in mice. Gut (2005) 54(12):1768–75. doi: 10.1136/gut.2005.067900 PMC1774778 16033879

[34] Yu Q Stamenkovic I. Angiopoietin-2 is implicated in the regulation of tumor angiogenesis. Am J Pathol (2001) 158(2):563–70. doi: 10.1016/S0002-9440(10)63998-3 PMC1850318 11159193

[35] Gilkes DM Bajpai S Chaturvedi P Wirtz D Semenza GL. Hypoxia-inducible factor 1 (HIF-1) promotes extracellular matrix remodeling under hypoxic conditions by inducing P4HA1, P4HA2, and PLOD2 expression in fibroblasts. J Biol Chem (2013) 288(15):10819–29. doi: 10.1074/jbc.M112.442939 PMC3624462 23423382

[36] Hielscher A Qiu C Porterfield J Smith Q Gerecht S. Hypoxia affects the structure of breast cancer cell-derived matrix to support angiogenic responses of endothelial cells. J Carcinog Mutagen (2013) Suppl 13:005. doi: 10.4172/2157-2518.S13-005 PMC3940068 24600535

[37] De Palma M Biziato D Petrova TV. Microenvironmental regulation of tumour angiogenesis. Nat Rev Cancer (2017) 17(8):457–74. doi: 10.1038/nrc.2017.51 28706266

[38] Ghosh K Thodeti CK Dudley AC Mammoto A Klagsbrun M Ingber DE. Tumor-derived endothelial cells exhibit aberrant rho-mediated mechanosensing and abnormal angiogenesis in vitro. Proc Natl Acad Sci USA (2008) 105(32):11305–10. doi: 10.1073/pnas.0800835105 PMC2516246 18685096

[39] Haas TL Davis SJ Madri JA. Three-dimensional type I collagen lattices induce coordinate expression of matrix metalloproteinases MT1-MMP and MMP-2 in microvascular endothelial cells. J Biol Chem (1998) 273(6):3604–10. doi: 10.1074/jbc.273.6.3604 9452488

[40] Charous SJ Stricklin GP Nanney LB Netterville JL Burkey BB. Expression of matrix metalloproteinases and tissue inhibitor of metalloproteinases in head and neck squamous cell carcinoma. Ann Otol Rhinol Laryngol (1997) 106(4):271–8. doi: 10.1177/000348949710600402 9109715

[41] Zheng H Takahashi H Murai Y Cui Z Nomoto K Niwa H. Expressions of MMP-2, MMP-9 and VEGF are closely linked to growth, invasion, metastasis and angiogenesis of gastric carcinoma. Anticancer Res (2006) 26(5A):3579–83. 17094486

[42] Rojiani MV Alidina J Esposito N Rojiani AM. Expression of MMP-2 correlates with increased angiogenesis in CNS metastasis of lung carcinoma. Int J Clin Exp Pathol (2010) 3(8):775–81. PMC2993228 21151391

[43] Ricard-Blum S Vallet SD. Fragments generated upon extracellular matrix remodeling: Biological regulators and potential drugs. Matrix Biol (2019) 75-76:170–89. doi: 10.1016/j.matbio.2017.11.005 29133183

[44] Leek RD Hunt NC Landers RJ Lewis CE Royds JA Harris AL. Macrophage infiltration is associated with VEGF and EGFR expression in breast cancer. J Pathol (2000) 190(4):430–6. doi: 10.1002/(SICI)1096-9896(200003)190:4<430::AID-PATH538>3.0.CO;2-6 10699991

[45] Sounni NE Devy L Hajitou A Frankenne F Munaut C Gilles C. MT1-MMP expression promotes tumor growth and angiogenesis through an up-regulation of vascular endothelial growth factor expression. FASEB J (2002) 16(6):555–64. doi: 10.1096/fj.01-0790com 11919158

[46] Ghosh S Basu M Roy SS. ETS-1 protein regulates vascular endothelial growth factor-induced matrix metalloproteinase-9 and matrix metalloproteinase-13 expression in human ovarian carcinoma cell line SKOV-3. J Biol Chem (2012) 287(18):15001–15. doi: 10.1074/jbc.M111.284034 PMC3340257 22270366

[47] Sudhan DR Rabaglino MB Wood CE Siemann DW. Cathepsin l in tumor angiogenesis and its therapeutic intervention by the small molecule inhibitor KGP94. Clin Exp Metastasis (2016) 33(5):461–73. doi: 10.1007/s10585-016-9790-1 PMC5378387 27055649

[48] Malla RR Gopinath S Gondi CS Alapati K Dinh DH Gujrati M. Cathepsin b and uPAR knockdown inhibits tumor-induced angiogenesis by modulating VEGF expression in glioma. Cancer Gene Ther (2011) 18(6):419–34. doi: 10.1038/cgt.2011.9 PMC3096680 21394106

[49] Pranjol MZ Gutowski N Hannemann M Whatmore J. The potential role of the proteases cathepsin d and cathepsin l in the progression and metastasis of epithelial ovarian cancer. Biomolecules (2015) 5(4):3260–79. doi: 10.3390/biom5043260 PMC4693277 26610586

[50] Pranjol MZI Gutowski NJ Hannemann M Whatmore JL. Cathepsin d non-proteolytically induces proliferation and migration in human omental microvascular endothelial cells *via* activation of the ERK1/2 and PI3K/AKT pathways. Biochim Biophys Acta Mol Cell Res (2018) 1865(1):25–33. doi: 10.1016/j.bbamcr.2017.10.005 29024694

[51] Pranjol MZI Gutowski NJ Hannemann M Whatmore JL. Cathepsin l induces proangiogenic changes in human omental microvascular endothelial cells *via* activation of the ERK1/2 pathway. Curr Cancer Drug Targets (2019) 19(3):231–42. doi: 10.2174/1568009618666180831123951 30173647

[52] Pranjol ZI Whatmore JL. Cathepsin d in the tumor microenvironment of breast and ovarian cancers. Adv Exp Med Biol (2020) 1259:1–16. doi: 10.1007/978-3-030-43093-1_1 32578168

[53] Winiarski BK Wolanska KI Rai S Ahmed T Acheson N Gutowski NJ. Epithelial ovarian cancer-induced angiogenic phenotype of human omental microvascular endothelial cells may occur independently of VEGF signaling. Transl Oncol (2013) 6(6):703–14. doi: 10.1593/tlo.13529 PMC3890705 24466373

[54] Winiarski BK Cope N Alexander M Pilling LC Warren S Acheson N. Clinical relevance of increased endothelial and mesothelial expression of proangiogenic proteases and VEGFA in the omentum of patients with metastatic ovarian high-grade serous carcinoma. Transl Oncol (2014) 7(2):267–76 e4. doi: 10.1016/j.tranon.2014.02.013 24913675 PMC4101350

[55] Pranjol MZI Zinovkin DA Maskell ART Stephens LJ Achinovich SL Los DM. Cathepsin l-induced galectin-1 may act as a proangiogenic factor in the metastasis of high-grade serous carcinoma. J Transl Med (2019) 17(1):216. doi: 10.1186/s12967-019-1963-7 31269957 PMC6610868

[56] Buck MR Karustis DG Day NA Honn KV Sloane BF. Degradation of extracellular-matrix proteins by human cathepsin b from normal and tumour tissues. Biochem J (1992) 282 Pt 1:273–8. doi: 10.1042/bj2820273 PMC1130919 1540143

[57] Berchem G Glondu M Gleizes M Brouillet JP Vignon F Garcia M. Cathepsin-d affects multiple tumor progression steps *in vivo*: proliferation, angiogenesis and apoptosis. Oncogene (2002) 21(38):5951–5. doi: 10.1038/sj.onc.1205745 12185597

[58] Krishnamachary B Berg-Dixon S Kelly B Agani F Feldser D Ferreira G. Regulation of colon carcinoma cell invasion by hypoxia-inducible factor 1. Cancer Res (2003) 63(5):1138–43. 12615733

[59] Afik R Zigmond E Vugman M Klepfish M Shimshoni E Pasmanik-Chor M. Tumor macrophages are pivotal constructors of tumor collagenous matrix. J Exp Med (2016) 213(11):2315–31. doi: 10.1084/jem.20151193 PMC5068227 27697834

[60] Chen Y McAndrews KM Kalluri R. Clinical and therapeutic relevance of cancer-associated fibroblasts. Nat Rev Clin Oncol (2021) 18(12):792–804. doi: 10.1038/s41571-021-00546-5 34489603 PMC8791784

[61] Broadway R Patel NM Hillier LE El-Briri A Korneva YS Zinovkin DA. Potential role of diabetes mellitus-associated T cell senescence in epithelial ovarian cancer omental metastasis. Life (Basel) (2021) 11(8). doi: 10.3390/life11080788 PMC8401827 34440532

[62] Li S Zhang Q Hong Y. Tumor vessel normalization: A window to enhancing cancer immunotherapy. Technol Cancer Res Treat (2020) 19:1533033820980116. doi: 10.1177/1533033820980116 33287656 PMC7727091

[63] Nagl L Horvath L Pircher A Wolf D. Tumor endothelial cells (TECs) as potential immune directors of the tumor microenvironment - new findings and future perspectives. Front Cell Dev Biol (2020) 8:766. doi: 10.3389/fcell.2020.00766 32974337 PMC7466447

[64] Amersfoort J Eelen G Carmeliet P. Immunomodulation by endothelial cells - partnering up with the immune system? Nat Rev Immunol (2022). doi: 10.1038/s41577-022-00694-4 PMC8920067 35288707

[65] Griffioen AW Damen CA Blijham GH Groenewegen G. Tumor angiogenesis is accompanied by a decreased inflammatory response of tumor-associated endothelium. Blood (1996) 88(2):667–73. doi: 10.1182/blood.V88.2.667.bloodjournal882667 8695814

[66] Huang H Langenkamp E Georganaki M Loskog A Fuchs PF Dieterich LC. VEGF suppresses T-lymphocyte infiltration in the tumor microenvironment through inhibition of NF-kappaB-induced endothelial activation. FASEB J (2015) 29(1):227–38. doi: 10.1096/fj.14-250985 25361735

[67] Griffioen AW Damen CA Mayo KH Barendsz-Janson AF Martinotti S Blijham GH. Angiogenesis inhibitors overcome tumor induced endothelial cell anergy. Int J Cancer (1999) 80(2):315–9. doi: 10.1002/(SICI)1097-0215(19990118)80:2<315::AID-IJC23>3.0.CO;2-L 9935216

[68] Lopatina T Favaro E Danilova L Fertig EJ Favorov AV Kagohara LT. Extracellular vesicles released by tumor endothelial cells spread immunosuppressive and transforming signals through various recipient cells. Front Cell Dev Biol (2020) 8:698. doi: 10.3389/fcell.2020.00698 33015029 PMC7509153

[69] Hou ZH Xu XW Fu XY Zhou LD Liu SP Tan DM. Long non-coding RNA MALAT1 promotes angiogenesis and immunosuppressive properties of HCC cells by sponging miR-140. Am J Physiol Cell Physiol (2020) 318(3):C649–C63. doi: 10.1152/ajpcell.00510.2018 31693399

[70] Li Y Zhang X Zheng Q Zhang Y Ma Y Zhu C. YAP1 inhibition in HUVECs is associated with released exosomes and increased hepatocarcinoma invasion and metastasis. Mol Ther Nucleic Acids (2020) 21:86–97. doi: 10.1016/j.omtn.2020.05.021 32516736 PMC7281784

[71] Wang K Jiang Z Webster KA Chen J Hu H Zhou Y. Enhanced cardioprotection by human endometrium mesenchymal stem cells driven by exosomal MicroRNA-21. Stem Cells Transl Med (2017) 6(1):209–22. doi: 10.5966/sctm.2015-0386 PMC5442741 28170197

[72] Njock MS Cheng HS Dang LT Nazari-Jahantigh M Lau AC Boudreau E. Endothelial cells suppress monocyte activation through secretion of extracellular vesicles containing antiinflammatory microRNAs. Blood (2015) 125(20):3202–12. doi: 10.1182/blood-2014-11-611046 PMC4440888 25838349

[73] Gorter A Zijlmans HJ van Gent H Trimbos JB Fleuren GJ Jordanova ES. Versican expression is associated with tumor-infiltrating CD8-positive T cells and infiltration depth in cervical cancer. Mod Pathol (2010) 23(12):1605–15. doi: 10.1038/modpathol.2010.154 20729814

[74] Hwang I Kim JW Ylaya K Chung EJ Kitano H Perry C. Tumor-associated macrophage, angiogenesis and lymphangiogenesis markers predict prognosis of non-small cell lung cancer patients. J Transl Med (2020) 18(1):443. doi: 10.1186/s12967-020-02618-z 33228719 PMC7686699

[75] Schaaf MB Garg AD Agostinis P. Defining the role of the tumor vasculature in antitumor immunity and immunotherapy. Cell Death Dis (2018) 9(2):115. doi: 10.1038/s41419-017-0061-0 29371595 PMC5833710

[76] Motz GT Santoro SP Wang LP Garrabrant T Lastra RR Hagemann IS. Tumor endothelium FasL establishes a selective immune barrier promoting tolerance in tumors. Nat Med (2014) 20(6):607–15. doi: 10.1038/nm.3541 PMC4060245 24793239

[77] Lei X Lei Y Li JK Du WX Li RG Yang J. Immune cells within the tumor microenvironment: Biological functions and roles in cancer immunotherapy. Cancer Lett (2020) 470:126–33. doi: 10.1016/j.canlet.2019.11.009 31730903

[78] Rahma OE Hodi FS. The intersection between tumor angiogenesis and immune suppression. Clin Cancer Res (2019) 25(18):5449–57. doi: 10.1158/1078-0432.CCR-18-1543 30944124

[79] Tian X Shen H Li Z Wang T Wang S. Tumor-derived exosomes, myeloid-derived suppressor cells, and tumor microenvironment. J Hematol Oncol (2019) 12(1):84. doi: 10.1186/s13045-019-0772-z 31438991 PMC6704713

[80] Pan Y Yu Y Wang X Zhang T. Tumor-associated macrophages in tumor immunity. Front Immunol (2020) 11:583084. doi: 10.3389/fimmu.2020.583084 33365025 PMC7751482

[81] Fu LQ Du WL Cai MH Yao JY Zhao YY Mou XZ. The roles of tumor-associated macrophages in tumor angiogenesis and metastasis. Cell Immunol (2020) 353:104119. doi: 10.1016/j.cellimm.2020.104119 32446032

[82] Giese MA Hind LE Huttenlocher A. Neutrophil plasticity in the tumor microenvironment. Blood (2019) 133(20):2159–67. doi: 10.1182/blood-2018-11-844548 PMC6524564 30898857

[83] Bernard H Garmy-Susini B Ainaoui N Van Den Berghe L Peurichard A Javerzat S. The p53 isoform, Delta133p53alpha, stimulates angiogenesis and tumour progression. Oncogene (2013) 32(17):2150–60. doi: 10.1038/onc.2012.242 22733133

[84] Babaei G Aliarab A Asghari Vostakolaei M Hotelchi M Neisari R Gholizadeh-Ghaleh Aziz S. Crosslink between p53 and metastasis: focus on epithelial-mesenchymal transition, cancer stem cell, angiogenesis, autophagy, and anoikis. Mol Biol Rep (2021) 48(11):7545–57. doi: 10.1007/s11033-021-06706-1 34519942

[85] Ravi R Mookerjee B Bhujwalla ZM Sutter CH Artemov D Zeng Q. Regulation of tumor angiogenesis by p53-induced degradation of hypoxia-inducible factor 1alpha. Genes Dev (2000) 14(1):34–44. doi: 10.1101/gad.14.1.34 10640274 PMC316350

[86] Yamakuchi M Lotterman CD Bao C Hruban RH Karim B Mendell JT. P53-induced microRNA-107 inhibits HIF-1 and tumor angiogenesis. Proc Natl Acad Sci USA (2010) 107(14):6334–9. doi: 10.1073/pnas.0911082107 PMC2851979 20308559

[87] Salvesen HB Akslen LA. Significance of tumour-associated macrophages, vascular endothelial growth factor and thrombospondin-1 expression for tumour angiogenesis and prognosis in endometrial carcinomas. Int J Cancer (1999) 84(5):538–43. doi: 10.1002/(SICI)1097-0215(19991022)84:5<538::AID-IJC17>3.0.CO;2-B 10502735

[88] Sundaram P Hultine S Smith LM Dews M Fox JL Biyashev D. p53-responsive miR-194 inhibits thrombospondin-1 and promotes angiogenesis in colon cancers. Cancer Res (2011) 71(24):7490–501. doi: 10.1158/0008-5472.CAN-11-1124 PMC3242824 22028325

[89] Xu Z Guo C Ye Q Shi Y Sun Y Zhang J. Endothelial deletion of SHP2 suppresses tumor angiogenesis and promotes vascular normalization. Nat Commun (2021) 12(1):6310. doi: 10.1038/s41467-021-26697-8 34728626 PMC8564544

[90] Kim IK Kim K Lee E Oh DS Park CS Park S. Sox7 promotes high-grade glioma by increasing VEGFR2-mediated vascular abnormality. J Exp Med (2018) 215(3):963–83. doi: 10.1084/jem.20170123 PMC5839752 29444818

[91] Zheng R Li F Li F Gong A. Targeting tumor vascularization: promising strategies for vascular normalization. J Cancer Res Clin Oncol (2021) 147(9):2489–505. doi: 10.1007/s00432-021-03701-8 PMC11802031 34148156

[92] Lugano R Ramachandran M Dimberg A. Tumor angiogenesis: causes, consequences, challenges and opportunities. Cell Mol Life Sci (2020) 77(9):1745–70. doi: 10.1007/s00018-019-03351-7 PMC7190605 31690961

[93] Ansari MJ Bokov D Markov A Jalil AT Shalaby MN Suksatan W. Cancer combination therapies by angiogenesis inhibitors; a comprehensive review. Cell Commun Signal (2022) 20(1):49. doi: 10.1186/s12964-022-00838-y 35392964 PMC8991477

[94] Alard E Butnariu AB Grillo M Kirkham C Zinovkin DA Newnham L. Advances in anti-cancer immunotherapy: Car-T cell, checkpoint inhibitors, dendritic cell vaccines, and oncolytic viruses, and emerging cellular and molecular targets. Cancers (Basel) (2020) 12(7). doi: 10.3390/cancers12071826 PMC7408985 32645977

[95] Ren S Xiong X You H Shen J Zhou P. The combination of immune checkpoint blockade and angiogenesis inhibitors in the treatment of advanced non-small cell lung cancer. Front Immunol (2021) 12:689132. doi: 10.3389/fimmu.2021.689132 34149730 PMC8206805

[96] Liu YQ Wang XL He DH Cheng YX. Protection against chemotherapy- and radiotherapy-induced side effects: A review based on the mechanisms and therapeutic opportunities of phytochemicals. Phytomedicine (2021) 80:153402. doi: 10.1016/j.phymed.2020.153402 33203590

[97] Hurwitz H Fehrenbacher L Novotny W Cartwright T Hainsworth J Heim W. Bevacizumab plus irinotecan, fluorouracil, and leucovorin for metastatic colorectal cancer. N Engl J Med (2004) 350(23):2335–42. doi: 10.1056/NEJMoa032691 15175435

[98] Lopes-Coelho F Martins F Pereira SA Serpa J. Anti-angiogenic therapy: Current challenges and future perspectives. Int J Mol Sci (2021) 22(7). doi: 10.3390/ijms22073765 PMC8038573 33916438

[99] Bogusz J Majchrzak A Medra A Cebula-Obrzut B Robak T Smolewski P. Mechanisms of action of the anti-VEGF monoclonal antibody bevacizumab on chronic lymphocytic leukemia cells. Postepy Hig Med Dosw (Online) (2013) 67:107–18. doi: 10.5604/17322693.1038349 23475487

[100] Dickson PV Hamner JB Sims TL Fraga CH Ng CY Rajasekeran S. Bevacizumab-induced transient remodeling of the vasculature in neuroblastoma xenografts results in improved delivery and efficacy of systemically administered chemotherapy. Clin Cancer Res (2007) 13(13):3942–50. doi: 10.1158/1078-0432.CCR-07-0278 17606728

[101] Leslie KK Filiaci VL Mallen AR Thiel KW Devor EJ Moxley K. Mutated p53 portends improvement in outcomes when bevacizumab is combined with chemotherapy in advanced/recurrent endometrial cancer: An NRG oncology study. Gynecol Oncol (2021) 161(1):113–21. doi: 10.1016/j.ygyno.2021.01.025 PMC7994192 33541735

[102] Bi J Dixit G Zhang Y Devor EJ Losh HA Newtson AM. Advantages of tyrosine kinase anti-angiogenic cediranib over bevacizumab: Cell cycle abrogation and synergy with chemotherapy. Pharm (Basel) (2021) 14(7). doi: 10.3390/ph14070682 PMC8308742 34358108

[103] Qin S Li A Yi M Yu S Zhang M Wu K. Recent advances on anti-angiogenesis receptor tyrosine kinase inhibitors in cancer therapy. J Hematol Oncol (2019) 12(1):27. doi: 10.1186/s13045-019-0718-5 30866992 PMC6417086

[104] Raymond E Dahan L Raoul JL Bang YJ Borbath I Lombard-Bohas C. Sunitinib malate for the treatment of pancreatic neuroendocrine tumors. N Engl J Med (2011) 364(6):501–13. doi: 10.1056/NEJMoa1003825 21306237

[105] Manzat Saplacan RM Balacescu L Gherman C Chira RI Craiu A Mircea PA. The role of PDGFs and PDGFRs in colorectal cancer. Mediators Inflammation (2017) 2017:4708076. doi: 10.1155/2017/4708076 PMC5259650 28163397

[106] Klasa-Mazurkiewicz D Jarzab M Milczek T Lipinska B Emerich J. Clinical significance of VEGFR-2 and VEGFR-3 expression in ovarian cancer patients. Pol J Pathol (2011) 62(1):31–40. 21574104

[107] Huang D Ding Y Li Y Luo WM Zhang ZF Snider J. Sunitinib acts primarily on tumor endothelium rather than tumor cells to inhibit the growth of renal cell carcinoma. Cancer Res (2010) 70(3):1053–62. doi: 10.1158/0008-5472.CAN-09-3722 20103629

[108] Diaz R Nguewa PA Redrado M Manrique I Calvo A. Sunitinib reduces tumor hypoxia and angiogenesis, and radiosensitizes prostate cancer stem-like cells. Prostate (2015) 75(11):1137–49. doi: 10.1002/pros.22980 25893276

[109] Salehzahi F Tse J Lee J Selvaraj J. A method to obtain correct standard uptake values in pinnacle treatment planning system for target volume delineation. J Med Phys (2016) 41(4):240–5. doi: 10.4103/0971-6203.195188 PMC5228047 28144116

[110] Michaelson MD McKay RR Werner L Atkins MB Van Allen EM Olivier KM. Phase 2 trial of sunitinib and gemcitabine in patients with sarcomatoid and/or poor-risk metastatic renal cell carcinoma. Cancer (2015) 121(19):3435–43. doi: 10.1002/cncr.29503 26058385

[111] Crown JP Dieras V Staroslawska E Yardley DA Bachelot T Davidson N. Phase III trial of sunitinib in combination with capecitabine versus capecitabine monotherapy for the treatment of patients with pretreated metastatic breast cancer. J Clin Oncol (2013) 31(23):2870–8. doi: 10.1200/JCO.2012.43.3391 23857972

[112] Rizzo M Porta C. Sunitinib in the treatment of renal cell carcinoma: an update on recent evidence. Ther Adv Urol (2017) 9(8):195–207. doi: 10.1177/1756287217713902 29662544 PMC5896861

[113] Yadav K Lim J Choo J Ow SGW Wong A Lee M. Immunohistochemistry study of tumor vascular normalization and anti-angiogenic effects of sunitinib versus bevacizumab prior to dose-dense doxorubicin/cyclophosphamide chemotherapy in HER2-negative breast cancer. Breast Cancer Res Treat (2022) 192:131–42. doi: 10.1007/s10549-021-06470-7 PMC8841320 34928481

[114] Sorensen AG Batchelor TT Zhang WT Chen PJ Yeo P Wang M. A "vascular normalization index" as potential mechanistic biomarker to predict survival after a single dose of cediranib in recurrent glioblastoma patients. Cancer Res (2009) 69(13):5296–300. doi: 10.1158/0008-5472.CAN-09-0814 PMC2824172 19549889

[115] Tolaney SM Boucher Y Duda DG Martin JD Seano G Ancukiewicz M. Role of vascular density and normalization in response to neoadjuvant bevacizumab and chemotherapy in breast cancer patients. Proc Natl Acad Sci USA (2015) 112(46):14325–30. doi: 10.1073/pnas.1518808112 PMC4655544 26578779

[116] Liu D Li G Avella DM Kimchi ET Kaifi JT Rubinstein MP. Sunitinib represses regulatory T cells to overcome immunotolerance in a murine model of hepatocellular cancer. Oncoimmunology (2017) 7(1):e1372079. doi: 10.1080/2162402X.2017.1372079 29296523 PMC5739555

[117] Batchelor TT Mulholland P Neyns B Nabors LB Campone M Wick A. Phase III randomized trial comparing the efficacy of cediranib as monotherapy, and in combination with lomustine, versus lomustine alone in patients with recurrent glioblastoma. J Clin Oncol (2013) 31(26):3212–8. doi: 10.1200/JCO.2012.47.2464 PMC4021043 23940216

[118] Robertson JD Botwood NA Rothenberg ML Schmoll HJ. Phase III trial of FOLFOX plus bevacizumab or cediranib (AZD2171) as first-line treatment of patients with metastatic colorectal cancer: HORIZON III. Clin Colorect Cancer (2009) 8(1):59–60. doi: 10.3816/CCC.2009.n.010 19203899

[119] Laurie SA Solomon BJ Seymour L Ellis PM Goss GD Shepherd FA. Randomised, double-blind trial of carboplatin and paclitaxel with daily oral cediranib or placebo in patients with advanced non-small cell lung cancer: NCIC clinical trials group study BR29. Eur J Cancer (2014) 50(4):706–12. doi: 10.1016/j.ejca.2013.11.032 24360368

[120] Ledermann JA Embleton AC Raja F Perren TJ Jayson GC Rustin GJS. Cediranib in patients with relapsed platinum-sensitive ovarian cancer (ICON6): a randomised, double-blind, placebo-controlled phase 3 trial. Lancet (2016) 387(10023):1066–74. doi: 10.1016/S0140-6736(15)01167-8 27025186

[121] Ledermann JA Embleton-Thirsk AC Perren TJ Jayson GC Rustin GJS Kaye SB. Cediranib in addition to chemotherapy for women with relapsed platinum-sensitive ovarian cancer (ICON6): overall survival results of a phase III randomised trial. ESMO Open (2021) 6(2):100043. doi: 10.1016/j.esmoop.2020.100043 33610123 PMC7903311

[122] Liu JF Brady MF Matulonis UA Miller A Kohn EC Swisher EM. Olaparib with or without cediranib versus platinum-based chemotherapy in recurrent platinum-sensitive ovarian cancer (NRG-GY004): A randomized, open-label, phase III trial. J Clin Oncol (2022) 40(19):2138–47. doi: 10.1200/JCO.21.02011 PMC9242406 35290101

[123] Itatani Y Kawada K Yamamoto T Sakai Y. Resistance to anti-angiogenic therapy in cancer-alterations to anti-VEGF pathway. Int J Mol Sci (2018) 19(4). doi: 10.3390/ijms19041232 PMC5979390 29670046

[124] He M Yang H Shi H Hu Y Chang C Liu S. Sunitinib increases the cancer stem cells and vasculogenic mimicry formation *via* modulating the lncRNA-ECVSR/ERbeta/Hif2-alpha signaling. Cancer Lett (2022) 524:15–28. doi: 10.1016/j.canlet.2021.08.028 34461182

[125] Tijeras-Raballand A Santos CD Serova M Martinet M Faivre S de Gramont A. Abstract 3000: Angiogenic recovery under chronic exposure to sunitinib is associated with vasculogenic mimicry in renal cell carcinoma. Cancer Res (2014) 74:3000. doi: 10.1158/1538-7445.AM2014-3000

[126] Liu K Zhang X Xu W Chen J Yu J Gamble JR. Targeting the vasculature in hepatocellular carcinoma treatment: Starving versus normalizing blood supply. Clin Transl Gastroenterol (2017) 8(6):e98. doi: 10.1038/ctg.2017.28 28617447 PMC5518951

[127] Yang J Liao C Liu Y Yang G Ke T Ding Y. MR imaging biomarkers evaluating vascular normalization window after anti-vessel treatment. Oncotarget (2018) 9(15):11964–76. doi: 10.18632/oncotarget.22600 PMC5844721 29552285

[128] Du R Lu KV Petritsch C Liu P Ganss R Passegue E. HIF1alpha induces the recruitment of bone marrow-derived vascular modulatory cells to regulate tumor angiogenesis and invasion. Cancer Cell (2008) 13(3):206–20. doi: 10.1016/j.ccr.2008.01.034 PMC2643426 18328425

[129] Haibe Y Kreidieh M El Hajj H Khalifeh I Mukherji D Temraz S. Resistance mechanisms to anti-angiogenic therapies in cancer. Front Oncol (2020) 10:221. doi: 10.3389/fonc.2020.00221 32175278 PMC7056882

[130] Harris L Batist G Belt R Rovira D Navari R Azarnia N. Liposome-encapsulated doxorubicin compared with conventional doxorubicin in a randomized multicenter trial as first-line therapy of metastatic breast carcinoma. Cancer (2002) 94(1):25–36. doi: 10.1002/cncr.10201 11815957

[131] Shafei A El-Bakly W Sobhy A Wagdy O Reda A Aboelenin O. A review on the efficacy and toxicity of different doxorubicin nanoparticles for targeted therapy in metastatic breast cancer. BioMed Pharmacother (2017) 95:1209–18. doi: 10.1016/j.biopha.2017.09.059 28931213

[132] Shi Y van der Meel R Chen X Lammers T. The EPR effect and beyond: Strategies to improve tumor targeting and cancer nanomedicine treatment efficacy. Theranostics (2020) 10(17):7921–4. doi: 10.7150/thno.49577 PMC7359085 32685029

[133] Wu J. The enhanced permeability and retention (EPR) effect: The significance of the concept and methods to enhance its application. J Pers Med (2021) 11(8). doi: 10.3390/jpm11080771 PMC8402171 34442415

[134] Dvorak AM Kohn S Morgan ES Fox P Nagy JA Dvorak HF. The vesiculo-vacuolar organelle (VVO): a distinct endothelial cell structure that provides a transcellular pathway for macromolecular extravasation. J Leukoc Biol (1996) 59(1):100–15. doi: 10.1002/jlb.59.1.100 8558058

[135] Huang N Liu Y Fang Y Zheng S Wu J Wang M. Gold nanoparticles induce tumor vessel normalization and impair metastasis by inhibiting endothelial Smad2/3 signaling. ACS Nano (2020) 14(7):7940–58. doi: 10.1021/acsnano.9b08460 32413258

[136] Darweesh RS Ayoub NM Nazzal S. Gold nanoparticles and angiogenesis: molecular mechanisms and biomedical applications. Int J Nanomed (2019) 14:7643–63. doi: 10.2147/IJN.S223941 PMC6756918 31571869

[137] Pan F Li W Yang W Yang XY Liu S Li X. Anterior gradient 2 as a supervisory marker for tumor vessel normalization induced by anti-angiogenic treatment. Oncol Lett (2018) 16(3):3083–91. doi: 10.3892/ol.2018.8996 PMC6096224 30127899

[138] Tabish TA Pranjol MZI Horsell DW Rahat AAM Whatmore JL Winyard PG. Graphene oxide-based targeting of extracellular cathepsin d and cathepsin l as a novel anti-metastatic enzyme cancer therapy. Cancers (Basel) (2019) 11(3). doi: 10.3390/cancers11030319 PMC6468385 30845739

[139] Tabish TA Pranjol MZI Karadag I Horsell DW Whatmore JL Zhang S. Influence of luminescent graphene quantum dots on trypsin activity. Int J Nanomed (2018) 13:1525–38. doi: 10.2147/IJN.S155021 PMC5858831 29588582

[140] Tabish TA Narayan RJ. Mitochondria-targeted graphene for advanced cancer therapeutics. Acta Biomater (2021) 129:43–56. doi: 10.1016/j.actbio.2021.04.054 33965624

[141] Tabish TA Hamblin MR. Multivalent nanomedicines to treat COVID-19: A slow train coming. Nano Today (2020) 35:100962. doi: 10.1016/j.nantod.2020.100962 32922510 PMC7473256

[142] Oduk Y Zhu W Kannappan R Zhao M Borovjagin AV Oparil S. VEGF nanoparticles repair the heart after myocardial infarction. Am J Physiol Heart Circ Physiol (2018) 314(2):H278–H84. doi: 10.1152/ajpheart.00471.2017 PMC5867653 29101176

[143] Roger Y Sydow S Burmeister L Menzel H Hoffmann A. Sustained release of TGF-beta3 from polysaccharide nanoparticles induces chondrogenic differentiation of human mesenchymal stromal cells. Colloids Surf B Biointerf (2020) 189:110843. doi: 10.1016/j.colsurfb.2020.110843 32044676

[144] Sun B Hyun H Li LT Wang AZ. Harnessing nanomedicine to overcome the immunosuppressive tumor microenvironment. Acta Pharmacol Sin (2020) 41(7):970–85. doi: 10.1038/s41401-020-0424-4 PMC7470849 32424240

[145] Liang Q Zhou L Li Y Liu J Liu Y. Nano drug delivery system reconstruct tumour vasculature for the tumour vascular normalisation. J Drug Target (2022) 30(2):119–30. doi: 10.1080/1061186X.2021.1927056 33960252

